# Silver nanostar films for surface-enhanced Raman spectroscopy (SERS) of the pesticide imidacloprid

**DOI:** 10.1016/j.heliyon.2023.e14686

**Published:** 2023-03-20

**Authors:** Norhayati Abu Bakar, Joseph G. Shapter

**Affiliations:** a Australian Institute for Bioengineering and Nanotechnology, University of Queensland, St. Lucia, Brisbane, Queensland, 4072 Australia; b Institute of Microengineering and Nanoelectronic, Universiti Kebangsaan Malaysia, UKM Bangi, 43600, Selangor, Malaysia

**Keywords:** Pesticides, Self-assembly, Silver nanostars, Surface-enhanced Raman scattering

## Abstract

Strategies for synthetic control of anisotropic metal nanostructures have grown in recent years in part due to their great potential for application as surface-enhanced Raman scattering (SERS) sensing substrates. It has been shown that SERS using silver substrates is a powerful tool for identification and qualification of trace chemical analysis on the basis of their unique molecular vibrations. In this work, we synthesized star-shaped silver nanostructures and fabricated SERS substrates to use the SERS enhancement of the Raman signal to detect neonicotinoid pesticides. These silver nanostar substrates were prepared by assembling the nanostar particles on a glass substrate surface using a self-assembly technique with various layers of silver nanostars film. The silver nanostar distribution on the solid substrate surface was found to have good reproducibility, reusability and were a stable SERS substrate giving SERS enhancements for pesticide detection at concentrations as low as 10^−6^ mg/ml. The distribution of these silver nanostars on the surface allowed excellent reproducibility of the detection with a low relative standard derivation (RSD) of SERS intensity of 8%. This work potentially builds a platform for an ultrasensitive detector where samples can be probed with little to no pre-processing and a range of pollutants can be detected at very low levels.

## 1 Introduction

Surface-enhanced Raman scattering (SERS) is a highly sensitive technique that has been developed from conventional Raman spectroscopy to enhance the Raman intensities of molecules. The SERS enhancement increases the molecular signal by more than 10^3^ after the adsorption of molecules on plasmonic nanostructured surfaces. Technically, SERS occurs when conduction electrons of metal nanoparticles on the surface interact with electromagnetic radiation such as light waves, which can lead to an amplification of natural electronic oscillation. This phenomenon enhances the optical near fields to allow single molecule detection. Thus, the SERS technique is commonly used in chemistry and biology analysis because of the high sensitivity and the fact that the spectra of molecular vibrations provide a fingerprint to identify molecules [1,2]. This means that the spectra can be used to detect and identify unknowns present at a very low concentrations in many matrices.

Arguably the large intensification in SERS is influenced by the metal nanoparticle material, the nanoparticle geometry and the SERS substrate platform which can determine the overall SERS effect [[3], [4], [5]]. Consequently, research in synthetic control of anisotropic metal nanostructures has been reported in recent years in SERS sensing applications. Because the anisotropic structure of the particles are employed to get large SERS enhancement, silver nanostars was first synthesized and employed in SERS detection of probenecid in 2013 by Garcia-Leis' group [6]. Silver has been extensively studied in SERS applications as it was one of the first materials discovered to produce Raman scattering enhancement and generates much larger electromagnetic enhancements resulting in high sensitivity. The Oliveira group has proven that the silver nanostars on paper surfaces has given good sensitivity for SERS substrates to detect Rhodamine 6G compared to silver nanoparticles on paper surfaces [7]. Their morphology and composition of the silver nanostars have been further tailored during the synthesis to get a larger SERS effect for detection [8].

The development of simple and low cost fabrication methods for solid SERS substrates have been studied intensely to take advantage of the large SERS enhancement of spectroscopic signals which allows the detection of trace amounts of chemicals. The goal of much of the recent efforts has been to use simple methods to produce sensitive SERS substrates which can be used multiple times. Interestingly, these SERS substrates are promising for use in the field due to portability and the fact that assembling the nanomaterials on solid substrates helps stabilize the active components extending shelf life. The handling of solid SERS substrates outside research labs is accessible to most scientists or technicians. Thus, these substrates would improve the quality of handheld and portable monitoring systems for trace amounts of chemicals in forensic, food safety, security, medical and environmental applications [[9], [10], [11], [12]].

Many techniques have been introduced to fabricate simple SERS substrate on solid surfaces rather than using nanoparticle suspension for SERS measurements. Drop casting of the suspension on a substrate was commonly used but destabilization of the suspension can lead to aggregation of particles on substrate surface, resulting in unexpected particle shapes on the surface, lack of sensitivity and reproducibility and limited substrate lifetimes. Traditional techniques for SERS substrate production to fabricate metal surfaces with nanoscale features are electron beam lithography, in situ growth of the nanoparticles by thermal or seed-mediated and self-assembly [[13], [14], [15], [16]]. These techniques are relatively straightforward for the preparation however need sophisticated instruments and some templates are involved. Studies on monolayer films on an oil-water interface which are easily transferred onto a substrate surface using self assembly technique has been reported by the Reincke group [17]. Interestingly this technique can assemble the nanoparticles from their colloidal suspension on solid surface with nanoscale features and avoid uncontrolled coagulation on the surface.

Numerous approaches have been directed towards preparing silver nanostars as SERS substrate owing to use its larger electromagnetic enhancements into trace analyte detection especially pesticides. Many good publications have proven the SERS effect is a powerful tool for identification and qualification to identify pesticides contamination. Pesticides are widely used in the agricultural sector to improve the productivity and the quality of crops. Unfortunately, most chemical pesticides pose risks to humans and the environment due to their essential toxicity to pests. The pesticides imidacloprid were first introduced as systemic insecticides to the global market in the early 1990s and were then the most commonly used neonicotinoid worldwide [18,19]. The demand for imidacloprid has been rapidly increasing year by year because of their extreme effectiveness against sucking insects and some biting insects and its rain resistance. However, neonicotinoids are compounds of great concern due to their toxicity, persistence and high solubility. They persist for long periods of time in the environment and can accumulate and pass from one species to the next through the food chain. Imidacloprid has been shown to have the highest pesticide residue in water sources and soils with the pesticide detected in at 96.8% of the samples tested in Hainan, China using liquid chromatography-mass spectroscopy (LC-MS) and gas chromatography-mass spectroscopy (GC-MS) instruments [20]. Hence, the ability to monitor these imidacloprid concentration in the environment is critical as governments have regulated maximum residue limits. Silver flower [21] or gold coated silver flower [22] structures have been used to detect residual imidacloprid in milk or tea. In these cases, the deposition is done by simply dropping the appropriate dispersion on the substrate and drying in an oven.

In this work, we synthesized star-shaped nanostructures to induce SERS enhancement by getting localized surface plasmon resonances (LSPR) from the edge of silver nanostar particles. These silver nanostars (AgNs) were prepared by a direct chemical reduction technique at room temperature using two reduction agents. We studied the optical absorbance peak at 385 nm which contributed to the unique surface plasmon of the star-shaped structures which then gave a huge enhancement of the electromagnetic field at the interface between nanostars and analytes. The AgNs particles were 150–200 nm in average diameter from tip to tip with several arms. To implement detection, the AgNs colloid was deposited on the glass substrate surface via a layer by layer method using an aqueous – organic interface and the analyte was then dropped on the nanostars surface by a drop-casting technique. This AgNs substrate was found to be a good SERS-active substrate giving SERS enhancements during monitoring the residue of agricultural products. The distribution of these AgNs on the surface allowed excellent reproducibility of the detection with a low relative standard derivation of SERS intensity.

While nanostars are often used in chemical and pesticide analysis, the self-assembly method used in this work to prepare the SERS substrate has not been reported previously. Importantly, this approach ensures a high coverage while maintaining availability of the active sites on the nanostars. The substrate prepared in this work is a highly effective sensor that does not require complex sample preparation which is critical for real world use in analytical facilities or more importantly in the field by staff with little training.

## 2 Methodology

The materials for the synthesized colloidal silver nanostars (AgNs) are silver nitrate (≥99.0%, Sigma Aldrich), sodium hydroxide (≥98.0%, Chem-Supply Pty Ltd), hydroxylamine 50 wt % in water (Sigma Aldrich) and trisodium citrate dehydrate (≥99.0%, Sigma Aldrich). Toluene, (Analytical Reagent Grade, Chem-Supply Pty Ltd) and n,n-dimethylformamide (≥99.8%, Sigma Aldrich) were used to form an oil-water interface for the fabrication of monolayer AgNs films on silicon and glass surfaces. For SERS measurements, the neonicotinoid pesticide imidacloprid-pestanal (Analytical standard, Sigma-Aldrich) was used. These chemicals were used as received without any further purification.

Colloidal AgNs was prepared by a chemical reduction technique at room temperature as reported by the Garcia-Leis group [6]. The synthesis was started by mixing 2.0 ml of 6.0 × 10^- 2^ M hydroxylamine into 2.0 ml of 5 × 10^−2^ M sodium hydroxide. This solution was stirred for 1 min before 9.0 ml of 1 × 10^−3^ M silver nitrate solution was added into the solution. The mixture then stirred for 5 min and the solution color changed to dark grey. After that, 0.1 ml 1% w/v trisodium citrate dehydrate was added into the solution and the final color of the colloidal AgNs remained dark grey. The colloidal suspension was treated using an ultrasonic bath for 15 min and centrifuged at 6000 rpm for 15 min. The pellet on the bottom of the centrifuge tube was collected and used for further nanostar assembly onto solid surface as SERS substrates. The characterization of the optical absorbance and particle imaging of the colloidal AgNs were done using UV-2600 Shimadzu and Hitachi HT7700 120 kV TEM respectively.

AgNs films were prepared on silicon and glass surfaces using a self-assembly technique based on films formed at a colloidal aqueous-toluene interface as reported by the Zhang group [23]. To assemble AgNs on the solid surface, the monolayer film was prepared by mixing 12.0 ml colloidal AgNs and 6.0 ml toluene into a watch glass with diameter of 8.0 cm and 1.3 cm height. The water-oil interface then formed and clearly seen on the watch glass surface. After that, 2.0 ml of n,n-dimethylformamide (DMF) was added dropwise into the mixture. This mixture was left for 1 h to let the complete monolayer films form on the surface due to the effect of interfacial tension prior to carefully extracting the toluene from the surface. The monolayer AgNs films were transferred onto the solid substrate by placing the silicon or glass substrate surface under the formed monolayers solution and carefully pulling the substrate upwards at 90° to the interface using tweezers. The substrate was further dried in an oven at 50 °C - 60 °C for 40–60 min. The monolayer AgNs thin films were rinsed with copious ethanol and purged with nitrogen. This process was repeated multiple times to add multiple layers to the substrate. These steps were repeated to prepare 1 layer, 5 layers, 10 layers and 15 layers of AgNs films on the substrate surfaces to study the effect of surface coverage of the AgNs films on SERS performance. In this study, we assembled the nanostars on silicon substrates for SEM characterization and glass substrates for optical absorbance characterization and as a SERS substrate to sense neonicotinoid insecticide. These thin films were characterized using UV-2600 Shimadzu spectrometer for optical absorbance and JEOL JSM-7001F for surface imaging of the films surface.

The pesticide imidacloprid solution was prepared by diluting 1 mg in 0.5 ml methanol and this solution was stirred using ultrasound and 0.5 ml of deionized water was then added into the solution. This solution was used as a stock solution to make dilutions for various concentrations. Seven concentrations of imidacloprid solution was prepared by diluting in water for 1 × 10°, 1 × 10^−1^, 1 × 10^−2^, 1 × 10^−3^, 1 × 10^−4^, 1 × 10^−5^, 1 × 10^−6^ mg/ml. The pesticide solution was dropped onto the bare glass for reference and onto the silver nanostars thin films for SERS measurement using a 785 nm laser wavelength from a Renishaw Raman spectrometer. The Raman spectrum for imidacloprid on glass substrate surface as reference spectrum and SERS spectra of imidacloprid on silver nanostars were recorded using 50× objective at 1% of 167 mWatt power laser and 5 accumulations for each measurement. However, the Raman spectrum for imidacloprid powder was recorded with 5 accumulations using 50× objective at 100% of the 167 mWatt power laser.

## 3 Results and discussion

**Fig. 1:**
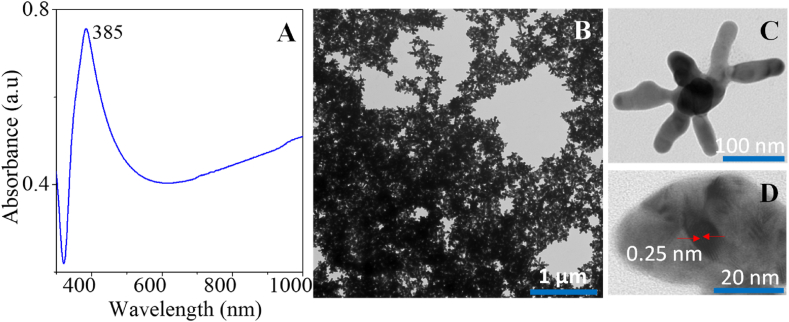
(A) Absorbance spectrum of the colloidal AgNs and (B–D) TEM images of nanostars on a carbon grid.

The colloidal silver nanostars (AgNs) were successfully synthesized after chemical reduction of Ag^+^ to Ag^0^ by hydroxylamine and trisodium citrate dehydrate at room temperature. The formation of the nanostars structure was clear during the synthesis due to the change of the colorless solution to dark grey after 5 min of the Ag^+^ reduction. Fig. 1(A) shows the optical absorbance of the AgNs suspension with the strong plasmon band at 385 nm. These two resonances features confirm the existence of the AgNs with the different numbers of arms and different tip sharpnesses. This result is confirmed with TEM imaging shown in Fig. 1(B–D) clearly showing the abundance of the AgNs with different arm shapes and multiple branches and spikes, various tip and arm lengths. The average tip-to tip length of the nanostars was 180 nm and the number of arms varied per particle ranging from 6 to 12. The lattice spacing was measured at 0.25 nm which corresponds to the lattice spacing of face-centred cubic (FCC) silver crystal (Fig. 1(D)).

**Fig. 2:**
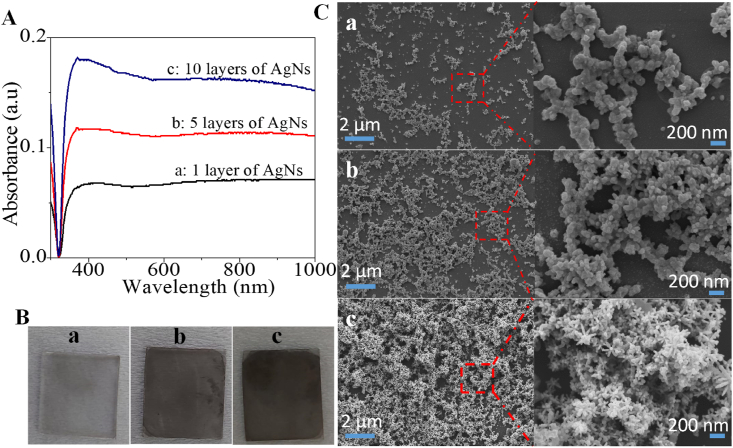
AgNs film characterization: (A) Optical absorbance (B) Photographs of the AgNs films surface on the glass substrates (C) SEM images: (a) 1 layer of AgNs film (b) 5 layers of AgNs film (c) 10 layers of AgNs films.

This colloidal suspension was used to fabricate the AgNs film on solid substrates via a self-assembly technique. The optical properties for 1 layer, 5 layers and 10 layers of AgNs films displayed a plasmon band at 380 nm in Fig. 2(A). This peak’s intensity aligned closely with the density and coverage of the AgNs on the substrate surface as seen from the photograph of the glass surfaces in Fig. 2(B) and SEM images in Fig. 2(C). The surface color was darker with increasing coverage after the nanostars form a dense coverage on the glass surface with multiple deposition of the nanoparticles. From SEM imaging, we found that AgNs clusters were present on the surface after 1 layer deposition and 5 layers deposition saw the nanostars particles fill the empty surface sites. As a result, the surface coverage of the nanostar particles on substrate surface for 1 layer to 5 layers increased from 30% to 60%. Further deposition of the nanostars saw the surface coverage increase to 90%. The nanostars clustered on the existing AgNs and preserved the presence of spikes at all coverages. The multiple layer films ultimately increased the availability of the spikes needed for SERS activity. The various AgNs films were then used to detect the imidacloprid insecticide by dropping and drying the imidacloprid solutions on the films.

**Fig. 3:**
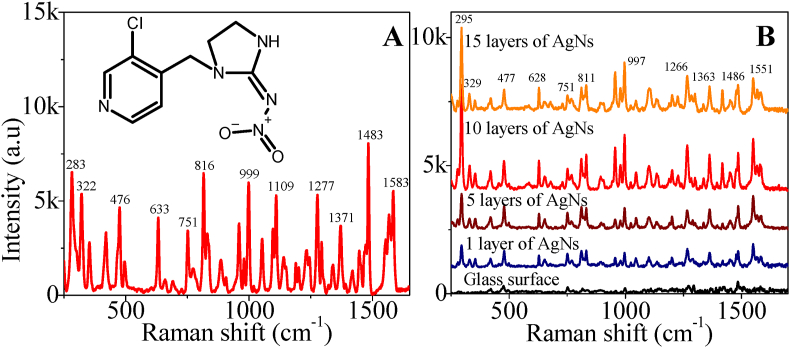
(A) Raman spectrum of the imidacloprid powder using 100% power laser and the chemical structure of the imidacloprid (B) SERS spectrum of imidacloprid on 1 layer, 5 layers, 10 layers and 15 layers of AgNs surface using 1% power laser. The spectrum on bare glass is provided for reference using 1% power laser.

The Raman spectra of imidacloprid powder and molecular formula are shown in Fig. 3(A). We observed the performance of the AgNs films as SERS substrates. The results are provided in Fig. 3(B) including imidacloprid on a bare glass surface for reference. In all cases, the concentration of the imidacloprid was 1 mg/ml. In the SERS spectra, the characteristic peaks of imidacloprid match those for imidacloprid on glass surface. The details of the characteristic peaks for imidacloprid are presented in the SI, Table S1. Impressively, the AgNs surface have dramatically enhanced the intensity of the characteristic peaks of imidacloprid compared to those observed on the bare glass. The intensity of the SERS spectra increased up to 10 nanostar layers but further deposition did not increase the intensity. This is not unexpected as the morphology of the 10 and 15 layers substrates is very similar meaning that the availability of the nanoparticle spikes will be similar (see SI, Fig. S1). One of the highest peaks at 295 cm^− 1^ which represented C–H rocking and C–Cl bending assignments was chosen to calculate the EF value for various layer of AgNs film towards SERS detection. The EF value for 1 layer, 5 layers, 10 layers and 15 layers is 6.18 × 10^3^, 9.65 × 10^3^, 2.86 × 10^4^ and 2.33 × 10^4^ respectively. The calculation details for EF value of 10 layers of AgNs is provided in SI.

**Fig. 4:**
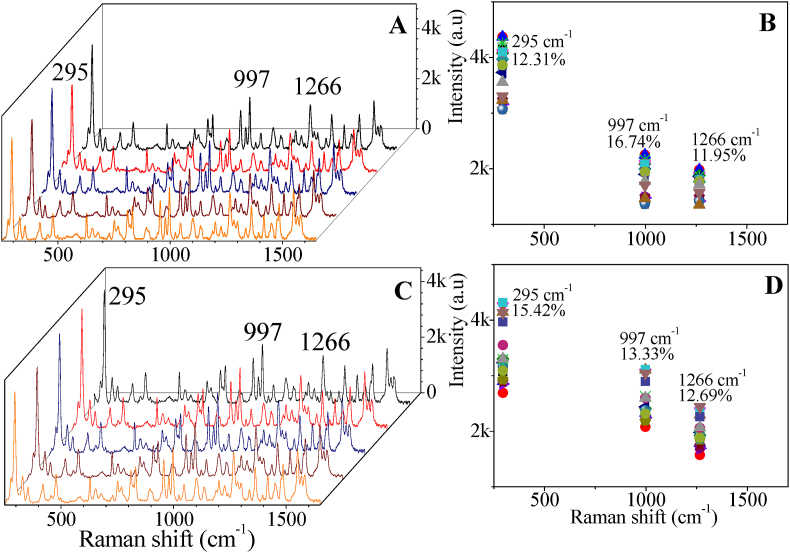
(A) SERS measurement of imidacloprid (1 mg/ml) for 5 different spots on the 10 layer substrate (a complete set of spectra from 20 spots is provided in the SI, Fig. S2(A)) (B) RSD for three peaks from the 20 different spots (C) SERS measurements for 5 different samples of imidacloprid (1 mg/ml) on 10 layers of AgNs surface (a complete set of spectra from 20 samples is provided in the SI, Fig. S2(B)) (D) RSD for 20 different samples of imidacloprid (1 mg/ml) on 10 layers of AgNs surface.

The reproducibility of the 10 layers substrate was explored by measuring SERS spectra from 20 different spots. The full SERS spectra of 20 different spots on same sample surface and 20 different sample surfaces are provided in the SI, Fig S2. The spectra were plotted in Fig. 3, Fig. 4 and 3 selected characteristic peaks have been chosen for relative standard derivative (RSD) percentage studied in Fig. 4(B). 10 layers of AgNs showed a good reproducibility for imidacloprid 1 mg/ml on the surface with the highest RSD being 16.74%. The reproducibility from sample to sample was also examined. 20 different samples of the 10 layers of AgNs substrates were tested showing very similar results with the highest RSD being 15.42%. The pesticide SERS spectra were plotted in Fig. 4(C) for 5 different samples of 10 layers of AgNs substrates and 3 selected characteristic peaks have been chosen for RSD percentage studied in Fig. 4(D). Ten layers of AgNs has created good local field enhancement for SERS detection as we observed the SERS intensity of imidacloprid absorbed on AgNs surface has major uniform enhancement. The uniformity of the nanostar distribution that formed from the cluster of AgNs and fully covered the substrate surface gives good SERS signal reproducibility. Moreover, after storage for 1 month in a small transparent container with silica gel, the AgNs films still show the same SERS intensity as newly prepared AgNs films. The stability SERS spectra of imidacloprid on 10 layers and the RSD for three characteristic peaks of imidacloprid are provided in SI, Fig. S3.

**Fig. 5:**
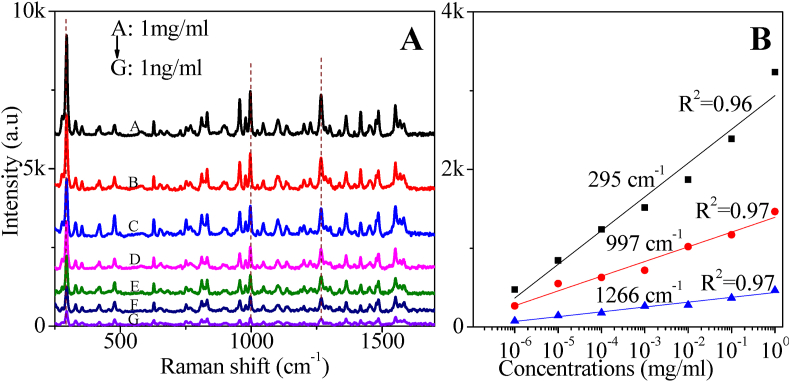
(A) SERS measurement for different concentrations of imidacloprid on 10 layers of AgNs (B) Log relationship graph for imidacloprid characteristic peaks versus concentrations.

We explored the use of the 10 layers of AgNs film for the qualitative detection of imidacloprid. Fig. 5(A) demonstrates that imidacloprid can be detected using the AgNs substrate at a concentration as low as 1 ng/ml by giving clear strong characteristic peaks. The SERS intensity decreased with decreasing imidacloprid concentrations on the AgNs surface as expected. We found that a log relationship between SERS intensity and concentrations of imidacloprid in Fig. 5(B) has good correlation as high as 0.97 for three characteristic peaks of imidacloprid [24]. Log relationships for SERS intensity to concentration have been observed previously. This result is good to apply in estimating the imidacloprid residue in food products as the result is significantly lower than the current allowed limit of residue in fruit by government China set at 1.91–3.91 mol/L (4.89 × 10^−4^ mg/ml-10.0 × 10^−4^ mg/ml) and by European Union at 0.5 mg/kg (5 × 10^−4^ mg/ml) [25]. The sensitivity observed using the AgNs is similar to that observed with the silver nanoflowers [21] but the addition of gold to the silver nanoflowers has been shown to improve the sensitivity by a factor of about 100 [22]. Other recent reports have demonstrated very similar limits of detection for imidacloprid using SERS substrates. In these cases the SERS substrates were either roughened silver [26] or palladium nanoparticles on meso-porous silicon [27]. These reports have checked reproducibility and sensitivity as expected. However, we have also checked the reusability of our substrate—that is using the same substrate multiple times. This assessment is critical for development of a sensor that will be used in the field typically by staff with little training.

**Fig. 6:**
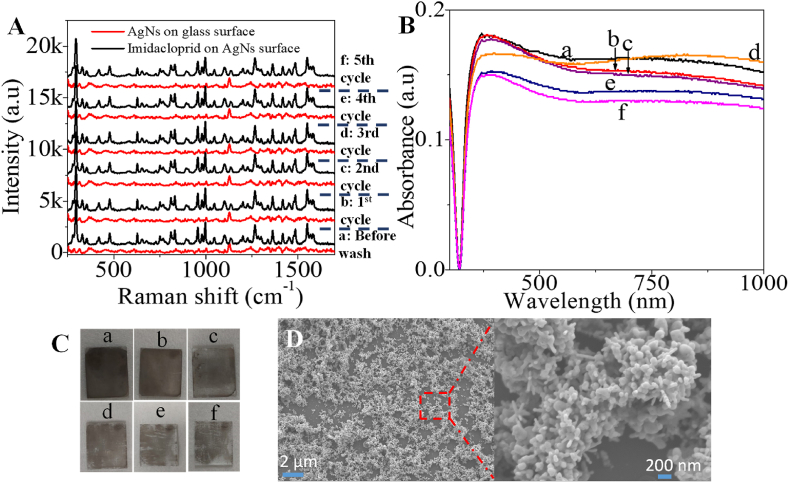
(A) Reusability performance of 10 layers of AgNs towards imidacloprid detection. (B) UV–Vis of AgNs substrate before wash to 5th cycle of washing (C) Photos of the surface of the AgNs substrate before wash (a) to 5th cycle (f) of washing (D) SEM image for AgNs surface after 5th cycle washing.

An important aspect of any detection system is its ability to be used for multiple tests. We observed the reusability of the 10 layers of AgNs film for imidacloprid detection. To study the reusability performance of the SERS substrate, Raman spectrum of AgNs films, SERS spectrum of imidacloprid on AgNs film and Raman spectrum of the AgNs after removal of imidacloprid on the AgNs surface were recorded for 5 cycles as displayed in Fig. 6 (A). The reusability procedure was done by using the SERS substrates as outlined and then cleaning by washing with ethanol to remove the adsorbed analyte. The removal of the analyte was confirmed by recording the Raman signal of the film. The same concentration of analyte was then added to the film surface and a SERS spectra of the pesticide was measured again. Over 5 cycles, there is very little degradation for the observed SERS signal confirming the robustness of the substrate. UV–Vis (Fig. 6(B)) and SEM (Fig. 6(D)) confirm that there are very few changes through the cycling process. From the photograph of the SERS substrate during washing in Fig. 6 (C), the color of the AgNs films surface fades after a few washings. Even though the optical images do show that there are some changes in the substrate, the thickness of the AgNs coverage means that the available active components are not changing much leading to consistent SERS performance. The absorbance intensity for the film surface confirms the consistency of the substrates with washing with slight decreases observed just for the last two cycles (Fig. 6(C)).

## 4 Conclusion

This work has shown that SERS using silver nanostars (AgNs) is a powerful tool for sensing the pesticide imidacloprid at very low concentrations. The AgNs film with a dense coverage of AgNs on a solid surface produced using a self-assembly shows the highest SERS enhancement towards the detection with an EF value of 2.86 × 10^4^ with a limit of detection of 10^−6^ mg/ml. The SERS-active substrates fabrication using this self-assembly technique to assemble AgNs on solid substrates allowed excellent reproducibility of the detection with a low relative standard derivation of SERS intensity. The substrate was shown to be reusable for several cycles for pesticide monitoring. Such reusability will be critical for development of handheld sensors to be used in the field with little or no high level technical support available.

## Author contribution statement

Norhayati Abu Bakar: Conceived and designed the experiments; Performed the experiments; Analyzed and interpreted the data; Wrote the paper.

Joseph G. Shapter: Conceived and designed the experiments; Analyzed and interpreted the data; Contributed reagents, materials, analysis tools or data.

## Funding statement

Joseph George Shapter was supported by 10.13039/501100000923Australian Research Council [DP200101217].

## Data availability statement

Data will be made available on request.

## Declaration of interest’s statement

The authors declare that they have no known competing financial interests or personal relationships that could have appeared to influence the work reported in this paper.
