# Supplementary material for: Silver nanostar films for surface-enhanced Raman spectroscopy (SERS) of the pesticide imidacloprid
Source: Heliyon. 2023 Mar 20;9(3):e14686. doi: 10.1016/j.heliyon.2023.e14686 (PMC10040700; doi:10.1016/j.heliyon.2023.e14686)
Supplement: Multimedia component 1 [file mmc1.docx]

Appendix A: Supporting Information:

Fig. S1. Characterization of AgNs film from 15 layers of deposition: (A) Optical absorbance with photograph of the AgNs films surface on the glass substrates (B) SEM images on silicon substrate.

The optical properties for 15 layers of AgNs films showed a plasmon band at 380 nm in Fig S1(A). The substrate surface was darker as seen from the photograph of the glass surfaces in inset Fig. S1(A) and dense cover of AgNs particles on silicon surface as shown in SEM images in Fig. S1(B).

The reproducibility parameter of the SERS detection of 1mg/ml imidacloprid was studied by taking 20 spots on the 1 sample of 10 layers AgNs film (Fig. S1(A)) and collecting one spot on 20 different samples of AgNs films (Fig. S1(B)). The relative standard derivation (RSD) was observed from 3 peaks of the highest SERS signal of imidacloprid and the RSD results were discussion in Results and Discussion section in this article.

Fig. S2. (A) SERS measurement of 1 mg/ml imidacloprid for 20 spots on the 10 layers AgNs substrate (B) SERS measurement for 20 different samples of 1 mg/ml imidacloprid on 10 layers AgNs substrate.

We observed the stability of the AgNs films towards detection of imidacloprid. This AgNs films were kept for 1 month in a small transparent container with silica gel to observe the SERS signal of imidacloprid. This container was left at room temperature. Based on the SERS of imidacloprid on AgNs surface on Fig. S3, the SERS spectra are almost identical and stable from 1 day to 1 month. The lowest RSD for three peak is 3.53%.

Fig. S3. (A) The stability of SERS signal of imidacloprid on 10 layers of AgNs films (B) RSD for three peaks from 1 day to 1 month.

The Raman spectra of imidacloprid powder, SERS of imidacloprid on AgNs surface and DFT of imidacloprid for each characteristic peak of imidacloprid were showed in Table S1. We observed the performance of the AgNs films as SERS substrates by studying all characteristic peaks of imidacloprid. The characteristic peaks of imidacloprid match those for imidacloprid powder and imidacloprid on various layers of the silver nanostars surfaces. The shifting of characteristic peaks for SERS signal of imidacloprid compared to Raman spectrum of imidacloprid powder and DFT is expected due to the existance of many matrix contributions from the AgNs substrate surface.

Table S1: The vibrational assignment for the characteristic peaks of imidacloprid powder, SERS signal of imidacloprid and DFT calculation.

| Experimentally observed peaks | | Density Funtional Theory (DFT) (cm^-1^) [1] | Assignments |
| --- | --- | --- | --- |
| Imidacloprid powder (cm^-1^) | SERS (cm^-1^) |  |  |
| 283 | 295 | 274 | C-H rocking, C-Cl bending |
| 322 | 329 | 306 | C-N wagging, C-N bending |
| 476 | 477 | 453 | C-N rocking, C-N bending, C-Cl stretching, C-C wagging, C-N twisting |
| 633 | 628 | 622 | C-N bending, C-C bending |
| 751 | 751 | 768 | N-O bending, C-N bending, C-C stretching, C-H rocking |
| 816 | 811 | 815 | C-N bending, C-C stretching, C-Cl stretching, C-H rocking |
| 999 | 997 | 1008 | C-N bending, C-N bending |
| 1109 | 1106 | 1126 | C-H bending, C-Cl stretching |
| 1142 | 1140 | 1197 | C-C bending, C-H wagging |
| 1190 | 1190 | 1217 | C-H twisting |
| 1245 | 1242 | 1254 | C-C twisting, C-H wagging, C-N stretching, N-N stretching |
| 1277 | 1266 | 1280 | N-O stretching, C-H twisting, C-H wagging, C-H bending |
| 1295 | 1300 | 1282 | C-H wagging, C-H bending, N-O stretching |
| 1371 | 1363 | 1376 | C-C stretching, C-N stretching, C-H wagging |
| 1448 | 1450 | 1449 | C-C stretching, C-N stretching, C-H bending |
| 1483 | 1486 | 1483 | C-H bending |
| 1551 | 1552 | 1547 | C-C stretching |
| 1583 | 1583 | 1606 | C-C stretching |

EF calculation:

There are a few enhancement factor (EF) formula for EF calculation and here we chose the Analytical Enhancement Factor approach as described by Ru et al. group [2]. These EF value was calculated using Formula 1.

$EF=\frac{I_{SERS}/N_{SERS}}{I_{Raman}/N_{Raman}}$ (1)

Where *I_Raman_*: Intensity of non-SERS; *I_SERS_*: Intensity of SERS; *N_Raman_*: Average number of molecule for non-SERS that contributed the signal and *N_SERS_*: Average number of molecule for SERS that contributed the signal

In this study, the deposition of pesticide on the AgNs surface was assumed as monolayer, so the Formula 1 can be derived to Formula 2.

$=\frac{I_{SERS}}{A\times\frac{M_{SERS}}{S_{SERF}}}\times\frac{A\times\frac{M_{Raman}}{S_{Raman}}}{I_{Raman}}$ (2)

Where *M_SERS_*: Number of dropped molecules on the glass surface for non-SERS, *M_Raman_*: Number of dropped molecule on the AgNs surface for SERS; *S_SERS_*: Geometry area of pesticide deposition for non-SERS; *S_Raman_*: Geometry area of pesticides for SERS and A is laser spot area.

The imidacloprid pesticide was dropped and dried on the glass and AgNs surface. The volume of imidacloprid is 0.08 ml on the surfaces. The glass substrate used for substrate at 1.2 cm x 1.2 cm measurement. So, the diameter of pesticide deposition after drying is 1.1 cm. This drying pesticide diameter for non-SERS and SERS surface is the same because the used substrates measurement in this work are same, so *S_SERS_* and *S_Raman_* value can be ignored. A can be also ignored due to same excitation angle and diameter of spot laser used for this Raman and SERS measurement. The final formula was derived to Formula 3.

$EF=\frac{I_{SERS}\times M{}_{Raman}}{I_{Raman}\times M_{SERS}}$ (3)

We used the most intense characteristic peak of imidacloprid at 295 cm^-1^ to calculate EF value. This example will use the Raman spectrum of imidacloprid on glass with concentration of imidacloprid at 1 g/ml (3.91x10^-2^ mol) because the charactristic peak at 1 mg/ml (3.91x10^- 6^ mol) is not clear to make comparison with SERS signal as shown in Fig. S4. SERS spectrum used in this calculation is imidacloprid on 10 layers of AgNs at 1 mg/ml (3.91x10^- 6^ mol). We also calculate EF value for imidacloprid on 1 layer, 5 layers and 15 layers AgNs surface using this formula.

The EF calculation for imidacloprid on 10 layer AgNs surface as described:

$$\begin{matrix} M_{Raman}=mol\times Avogadro's number \\ =3.91\times10^{-2}\times6.022\times10^{23} \\ =2.35\times10^{22} \end{matrix}$$

$$\begin{matrix} M_{SERS}=mol\times Avogadro's number \\ =3.91\times10^{-6}\times6.022\times10^{23} \\ =2.35\times10^{18} \end{matrix}$$

$$\begin{matrix} I_{SERS} =4145.33 \\ I_{Raman}=1450.14 \end{matrix}$$

$$EF=\frac{4145.33\times2.35\times10^{22}}{1450.14\times2.35\times10^{18}}$$

$$\begin{matrix} EF =2.86\times10^{4} \\ \end{matrix}$$

Fig. S4. Raman spectra of 1mg/ml and 1 g/ml imidacloprid on glass surface and SERS spectrum of 1 mg/ml imidacloprid on 10 layers AgNs surface.

References:

[1] N. Creedon, P. Lovera, J.G. Moreno, M. Nolan, A. O’Riordan, Highly Sensitive SERS Detection of Neonicotinoid Pesticides. Complete Raman Spectral Assignment of Clothianidin and Imidacloprid, J. Phys. Chem. A. 124 (2020) 7238–7247. https://doi.org/10.1021/acs.jpca.0c02832.

[2] E.C. Le Ru, E. Blackie, M. Meyer, P.G. Etchegoin, Surface Enhanced Raman Scattering Enhancement Factors:  A Comprehensive Study, J. Phys. Chem. C. 111 (2007) 13794–13803. https://doi.org/10.1021/jp0687908.
